# Nesfatin-1 Influences the Excitability of Glucosensing Neurons in the Dorsal Vagal Complex and Inhibits Food Intake

**DOI:** 10.1371/journal.pone.0098967

**Published:** 2014-06-06

**Authors:** Jing Dong, Hong-Zai Guan, Zheng-Yao Jiang, Xi Chen

**Affiliations:** 1 Department of Special Medicine, Medical College of Qingdao University, Qingdao, China; 2 Department of Physiology, Medical College of Qingdao University, Qingdao, China; INRA, France

## Abstract

Nesfatin-1 is a recently discovered metabolic peptide hormone that decreases food intake after lateral, third, or fourth brain ventricle; cisterna magna; or paraventricular nucleus (PVN) injection in ad libitum fed rats. Additional micro-injection studies will improve the understanding of how nesfatin-1 acts on the brain and define specific nuclei responsive to nesfatin-1, which will provide insight on its effects on food intake. We evaluated how nesfatin-1 injection into the dorsal vagal complex (DVC) modulates food intake response in rats during the dark phase. Consistent with previous observations, nesfatin-1-injected rats significantly reduced cumulative food intake over a 5-h period in rats. Chronic administration of nesfatin-1 into the DVC reduced body weight gain over a 10-day period. Because glucosensing neurons in the DVC are involved in glucoprivic feeding and homeostatic control of blood glucose, we examined the effect of nesfatin-1 on the excitability of DVC glucosensing neurons. Nesfatin-1 inhibited most of the glucose-inhibitory (GI) neurons and excited most of the glucose-excitatory (GE) neurons in the DVC. Current-clamp electrophysiology recordings from DVC glucosensing neurons in slice preparation showed that bath applied nesfatin-1(10 nM) increased the firing frequency of GE neurons and inhibited the firing rate of GI-neurons. Nesfatin-1 inhibited 88.9% (16/18) of gastric distension inhibitory (GD-INH) neurons and excited 76.2% (32/42) of gastric distension excitatory (GD-EXC) neurons. Thus, nesfatin-1 may control food intake by modulating the excitability of glucosensing neurons in the DVC.

## Introduction

Nesfatin-1, an 82-amino-acid peptide derived from the nucleobindin 2 (NUCB2), is emerging as a new regulator of food intake [1] and is expressed in several regions of the brain, including the paraventricular nucleus of the hypothalamus (PVN), supraoptic nucleus (SON), arcuate nucleus (ARC), lateral hypothalamic area (LHA), zona incerta, and the nucleus of the solitary tract (NTS), a medullary structure that plays an important role in the regulation of feeding [2], [3], [4], [5]. Acute intracerebroventricular (i.c.v) [6], [7], third (3v) [1], [8], or fourth brain ventricle (4v) [6], cisterna magna [6] injection of nesfatin-1 decreases food intake in rats and mice. Furthermore, treating animals with nesfatin-1-neutralizing antibodies markedly increases food consumption and weight gain, underscoring the tonic inhibitory action this peptide exerts over ingestive behaviors [1]. Stengel et al. [6] points out that additional micro-injection studies will help define additional specific nuclei in the hindbrain that respond to nesfatin-1, which will elucidate the effects of this metabolic peptide on food intake and digestive function. A recent study by Maejima et al. [8] shows that administering nesfatin-1 into the 3v induces c-Fos expression specifically in the PVN and NTS, and focal injection of nesfatin-1 into the PVN induces c-Fos expression in the NTS. This selective c-Fos induction indicates these nuclei are likely involved in anorectic signaling by nesfatin-1. Moreover, peripheral injection of the satiety peptide cholecystokinin (CCK) activates NUCB2/nesfatin-1 immunopositive cells in the PVN and NTS of rats [9]. Vagal afferents within the central nervous system synapse on the NTS located in the caudal brainstem. The NTS also receives input from the area postrema (AP), which has a high permeability of the blood-brain barrier and is sensitive to the blood-borne signals linked to ingested macronutrients [10]. Moreover, numerous structures, including hypothalamic nuclei and the medullary dorsal vagal complex (DVC) which is composed of 3 parts: the AP, NTS and dorsal motor nucleus of the vagus nerve (DMNV) belong to the brain vagal-regulatory pathway activated during hypoglycemia and contain a large proportion of nesfatin-1-expressing neurons [11]. Subgroups of hindbrain catecholamine neurons selectively activated by hypoglycemic stimuli play a significant role in feeding and hyperglycemic responses to a glucose deficit [12], [13], [14]. Glucosensing neurons alter their action potential frequency responding to the changes in extracellular glucose concentration and initiate the counterregulatory response to hypoglycemia. DVC contains two kinds of glucosensing neurons, glucose-excited (GE) neurons, which increase its activity in response to an increase of local glucose levels, and glucose-inhibited (GI) neurons, which decrease its activity in response to a rise of local glucose levels. Thus, nesfatin-1 may trigger physiological and hormonal counter-regulations observed in response to hypoglycemia by modulating the activity of glucosensing neurons in DVC. We have previously shown that the activity of the glucosensing neurons in the LHA, PVN, and the ventromedial nucleus (VMN) are modulated by the administration of nesfatin-1 [15]. In the present study, we injected nesfatin-1 into the DVC at the level of the hindbrain and assessed food intake response in rats during the dark phase. To clarify the mechanisms by which nesfatin-1 exerts its satiety-promoting actions, we examined the effect of nesfatin-1 on the excitability of DVC glucosensing neurons.

DVC also contains another different kind of neuron, named gastric distention (GD) sensitive neurons which change their firing rate responding to GD. GD may mimic the physiological effect occurring in the gastrointestinal (GI) system postprandially. Gastric distension has been shown to trigger stretch as well as tension mechanosensitive receptors that in turn relay their information via vagal and splanchnic nerves to the hindbrain and several other brain areas. GD-induced satiation can also be regulated by GI hormones. Therefore, we also investigated the nesfatin-1-induced excitability of the GD-sensitive neurons in the DVC.

## Materials and Methods

### 1. Animals

Male Wistar rats (280–330 g, Institute of Drug Control of Qingdao, China) were housed in temperature controlled animal room (22±2°C) with free access to food and water in a 12 h light/dark cycle for at least 2 weeks before starting the experiments. Rats were group housed (five in each cage) in standard cages (54 cm×38 cm×20 cm) with 3 cm –deep wood based litter on the solid floors and with a water bottle and food pellets on the cage top. After the operation, rats were allowed to recover in individual cages for one day before transfer to standard group housing. All animal protocols were approved by the Animal Care and Use Committee of Qingdao University in accordance with the National Institutes of Health guidelines.

### 2. Food intake and body weight gain

After being anesthetized with chloral hydrate (80 mg/ml, 0.5 ml/100 g, i.p.), animals were positioned in a stereotaxic apparatus (Narishige SN-3, Tokyo, Japan), with the incisor bar 3.3 mm below the center of ear bars and the skull exposed. A stainless steel cannula (26 gauge, 15 mm) was implanted into the DVC via a stereotaxic device. Stereotaxic coordinates of the DVC were obtained from Paxinos and Watson's brain atlas [17] and were 13.8 mm caudal to the bregma, 0.5 mm lateral to the middle line, and 7.5 mm below the surface of the skull. After fixing the cannula and sealing all openings in skull with dental acrylic, the incision was sutured and a 28-gauge obturator was placed in the cannula. After full recovery (7 days), rats were placed in metabolic cages (Feeding and Activity Analyser 47552-002, Ugo Basile, Italy) at least 3 days and supplyed with lab chow and tap water for adapting to the injection procedure.

On experiment days, food was withdrawn from the metabolic cages at 15:00. Nesfatin-1 (1-82, Phoenix Pharmaceuticals, Burlingame, CA, USA) or an equivalent volume of saline was injected using a microsyringe that extended 0.7 mm below the guide cannula. For all experiments, nesfatin-1(15, 25, or 50 pmol in 0.5 µl of volume) or saline was delivered to the nuclei parenchyma at the speed of 0.25 µl/min. At the end of the injection, the injector was remained in the guide cannula for another 5 min. The rats were then allowed to re-feed, and cumulative food intake was measured from 19:00 to 7:00 using electronic precision scales. The variation of the food intake was continuously monitored using Data Acquisition software 51800 (Feed-Drink Monitoring System Ver. 1.31, Ugo Basile, Italy). Food intake was monitored up to 12 h and calculated as g/300 g body weight [6]. Body weight were measured daily throughout the testing period.

After accomplishing all experiments, brains were removed and stored in a 4% paraformaldehyde solution. Injection sites were stained with pontamine sky blue and the brains were sectioned in the coronal plane at a thickness of 50 µm on a freezing microtome (Kryostat 1720, Leica, Germany) to confirm the location of the cannula. The data of rats whose injection site was located in the correct place were used in our study.

### 3. Electrophysiological recordings *in vivo*

The rats were anesthetized with urethane (1.0 g/kg, i.p.) and positioned in a stereotaxic apparatus. Supplemental anesthetics were given when needed. After removing a portion of the occipital bone and cerebellum, the brainstem was exposed. To improve stability for neuronal recording, the dura mater and arachnoid of the exposed medulla were carefully removed and covered with warm agar (3–4% in saline). A four-barrel glass microelectrode was broken to a tip diameter of 3–10 µm under the microscope and used for electrophysiological recording [15]. The recording glass microelectrode was filled with 0.5 M sodium acetate and 2% pontamine sky blue. The other three barrels were connected with a four-channel pressure injector (PM2000B, Micro Data Instrument, Inc. USA) and filled with a 5 mM glucose solution (pH 7.4), a 1.5×10^−8^ M nesfatin-1 solution, and a 0.9% NaCl solution, respectively. Drugs were ejected on the surface of firing cells with short pulse gas pressure (1500 ms, 5.0–15.0 psi) [15].

We localized DVC neurons 0.2–1.0 mm lateral from the midline, 1.0 mm rostral and 0.5 mm caudal from the obex, and 0.5–1.8 mm deep from dorsal surface of the medulla [17]. The recorded electrical signals were amplified and displayed on a Memory Oscilloscope (VC-11, Nihon Kohden). The analog signals were fed into a signal analyzer and computer, which incorporated a signal discriminator to allow unitary data to be stored on-line. GD was created using a latex balloon attached to polyethylene tubing (PE-240) surgically placed in the stomach [18]. After midline laparotomy, we used warm isotonic saline to wash gastric contents through a small incision in the fundus wall. GD was applied by increasing the volume of the balloon. Volumes of 1.5–2.0 ml were routinely used in our study, as inflating the balloon at a rate of ∼0.5 ml/s to 2 ml does not affect arterial blood pressure [18]. Distension was maintained at a constant volume for 10–30 s. The pylorus was ligatured to avoid duodenal reflux, which may affect gastric volume.

### 4. Slice preparation and electrophysiology

Coronal slices (300 µm) of the DVC were dissected from 10–15 day old Sprague-Dawley rats and cut in ice-cold cutting solution containing (in mM): NaCl, 124; NaHCO_3_, 26; KCl, 3; NaH_2_PO_4_, 1.3; MgSO_4_, 2; CaCl_2_, 0.5; and glucose, 25; bubbled with 95% O_2_ and 5% CO_2_. The slices were cultured at 32°C for 1 h and then maintained at room temperature (22–25°C) for use during a period of less than 5 h.

Brain slices were transferred to a recording chamber and continuously perfused with artificial cerebral spinal fluid (ACSF) at room temperature (22–25°C) at a flow rate of 1.5–2 ml/min. The recording ACSF buffer is similar to the cutting solution except for the Mg^2+^ (1.3 mM) and Ca^2+^ (2.4 mM). Recording pipettes (2–4 ΩM) were pulled from borosilicate glass capillaries with a horizontal puller (P-97, Sutter Instrument, Novato, CA, USA) and filled with intracellular solution consisting of the following (in mM): K-gluconate, 130; KCl, 5; EGTA, 0.6; HEPES, 10; MgCl_2_, 4; Na_2_ATP, 3; Na_3_GTP, 0.3; and Na_2_-phosphocreatine, 10; 290 mOsm, pH 7.3 with KOH in RNase-free water.

DVC neurons were visualized under infrared light using a differential interference contrast microscope (Nikon, Japan). Whole-cell patch clamp recording of DVC neurons was carried out to record spikes under current-clamp mode. Electrophysiological signals were amplified using a Multiclamp 700B amplifier (Molecular Devices, Union City, CA, USA), acquired with a Digital 1440A interface (Molecular Devices) in conjunction with a computer, and analyzed by pClamp10 (Molecular Devices).

### 5. Statistical analysis

Data are expressed as mean ± SEM. Food intake data and body weight gain after nesfatin-1 or saline injection were analyzed using two-way repeated-measures ANOVA. When computer graphs indicated that the modification of neuronal activity lasted more than 1 minute during and after the ejection of reagent, the mean discharge frequencies during this period (60 s) of modified activity and the 60 s immediately prior to treatment were compared using Student's t-test. Changes in neuronal firing frequencies were considered to be responses if they were statistically significant and exceeded 20%. Statistical significance was set at P<0.05.

## Results

### 1. Feeding responses to nesfatin-1 injection into the DVC

**Figure 1:**
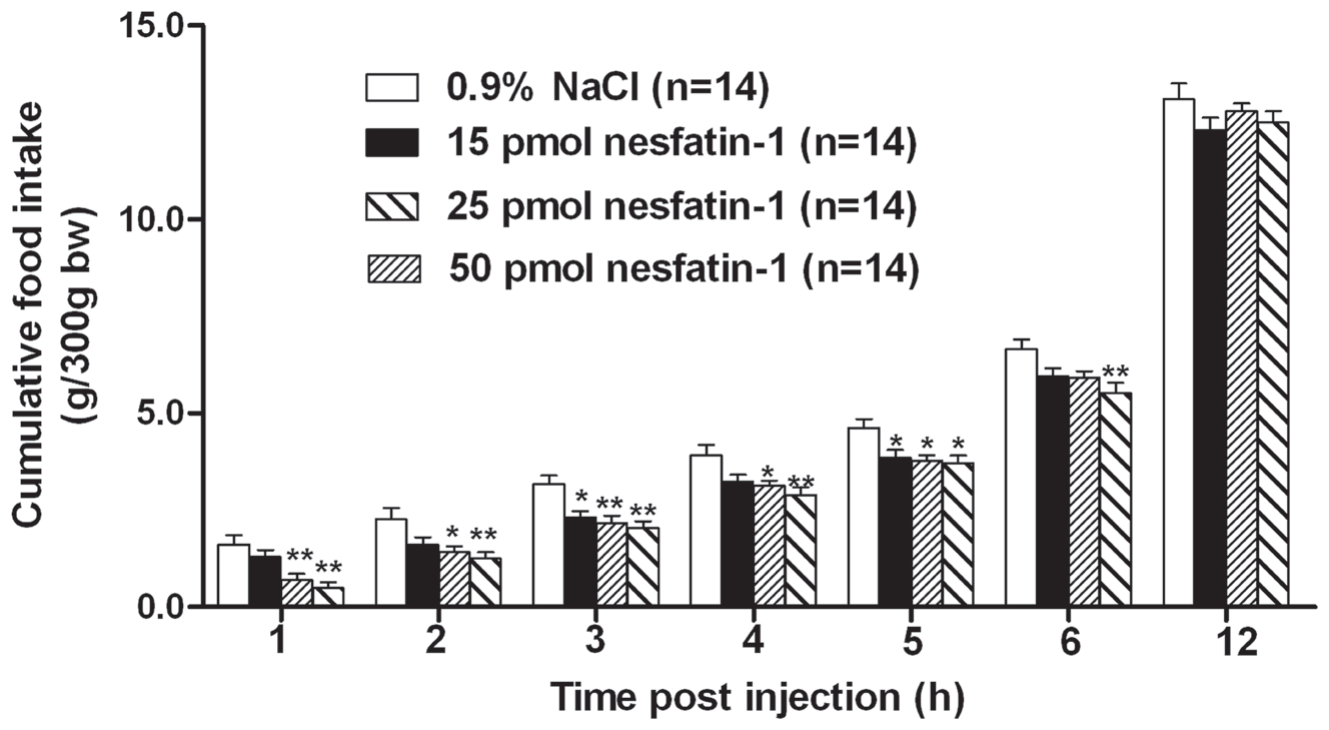
Nesfatin-1(15, 25, or 50 pmol) injected into the dorsal vagal complex (DVC) decreased dark phase food intake in rats. Cumulative food intake was monitored for 12±SEM. *P<0.05; **P<0.01 vs. vehicle.

Feeding response was tested in 65 rats, among which 9 were excluded due to misplacement of the cannula. All three doses (15, 25, and 50 pmol) of nesfatin-1 significantly decreased cumulative food intake (Fig.1). Rats administered 25 or 50 pmol nesfatin-1 consumed less food throughout the first 5 h of testing than their vehicle-treated counterparts. Rats administered 15 pmol nesfatin-1 ate less food at all time points compared to control, but this difference only reached significance 3 h and 5 h after injection. The 15, 25 and 50 pmol doses of nesfatin-1 induced 20.8%, 56.8% and 68.8% decreases in food intake, respectively, compared with their respective controls 1 h after injection. The 25 pmol nesfatin-1 injection into the DVC decreased cumulative food intake by 56.8%, 37.8%, 31.3%, 20.0% and 18.2%, respectively, compared with vehicle-injected controls at the 1, 2, 3, 4 and 5 h time points. Rats administered the highest dose tested (50 pmol nesfatin-1) consumed less food throughout the first 6 h of testing (vehicle vs. nesfatin-1, 1 h: 1.6±0.2 vs. 0.5±0.1 g/300 g body weight, P<0.01; 6 h: 6.7±0.3 vs. 5.5±0.3 g/300 g body weight, P<0.01) but no significant difference was observed between the 25 and 50 pmol nesfatin-1 groups.

**Figure 2:**
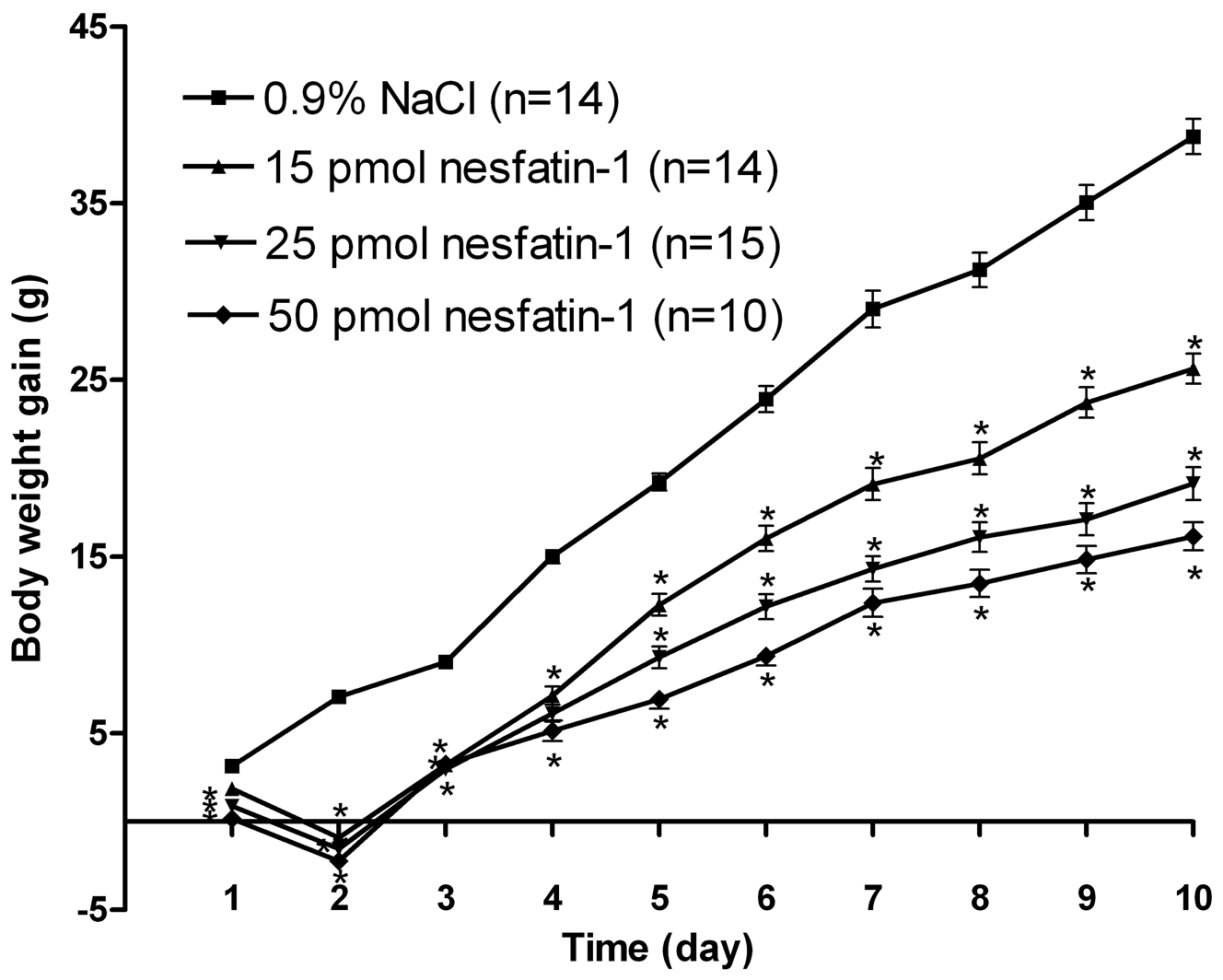
Change in body weight gain after continuous injection of nesfatin-1 for 10 days. Body weight gain (increment from day 0) in rats after nesfatin-1 injection (15, 25, or 50 pmol daily). Data are mean ± SEM *, P<0.01 compared with vehicle.

**Figure 3:**
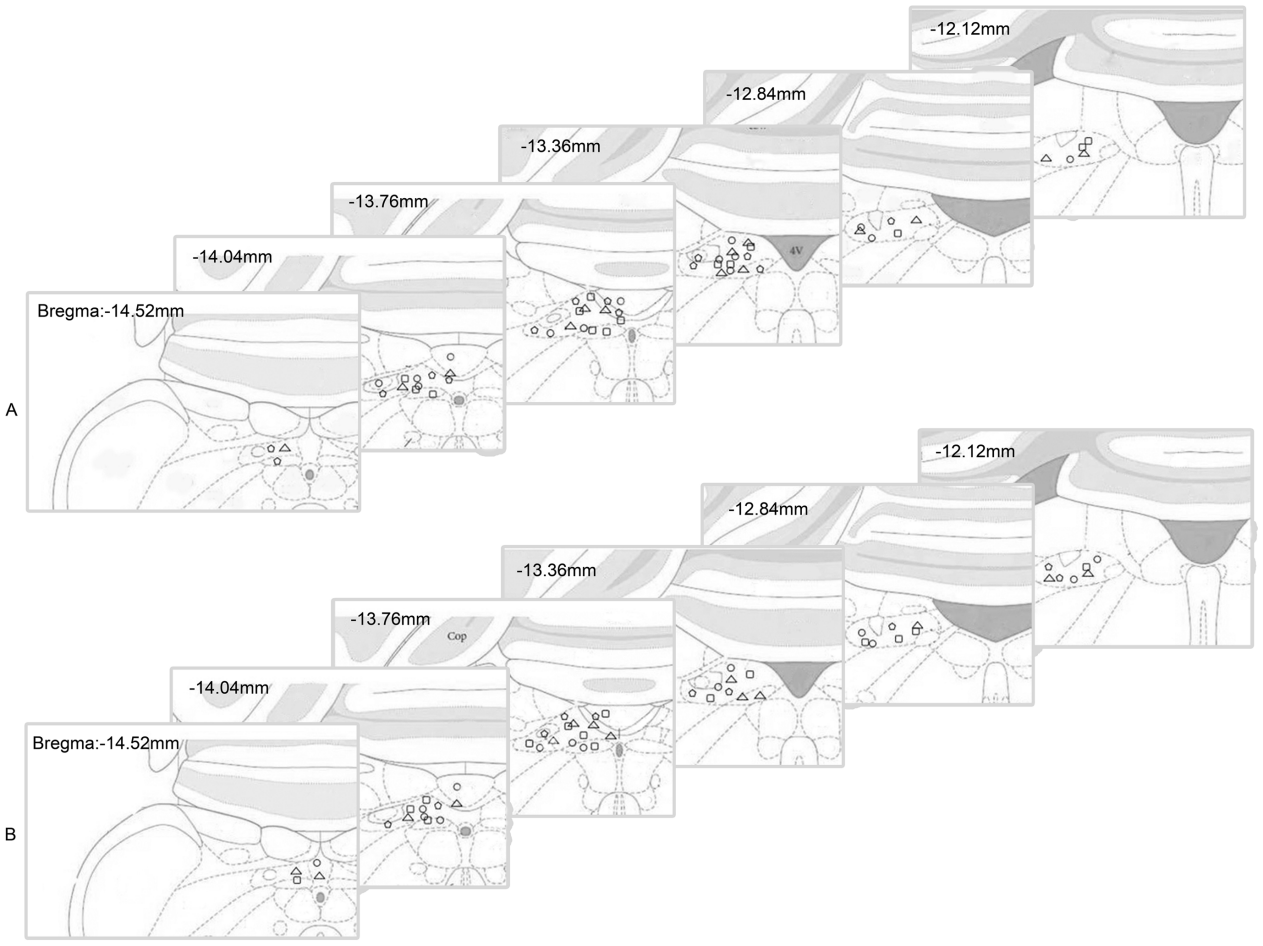
Maps of injection sites in DVC. A. Confirmation of injection sites in food intake measurement. Control (triangle, n = 14), 15 pmol nesfatin-1 (circle, n = 14), 25 pmol nesfatin-1 (square, n = 14), 50 pmol nesfatin-1 (pentagon, n = 14). B. Confirmation of injection sites in body weight gain measurement: control (triangle, n = 14), 15 pmol nesfatin-1 (circle, n = 14), 25 pmol nesfatin-1 (square, n = 15), 50 pmol nesfatin-1 (pentagon, n = 10).

To determine whether chronically administered nesfatin-1 caused any changes in body weight gain, 67 rats received an injection of either nesfatin-1 or saline into the DVC in the early dark phase once daily for 10 days. Among them, 14 rats were excluded due to misplacement of the cannula. Compared with saline-treated group, 15, 25, and 50 pmol nesfatin-1 injections reduced body weight gain in a dose-dependent manner (Fig. 2). Compared with the saline-treated group (n = 14), body weight gain was reduced by 34.0% (nesfatin-1-treated 25.6±0.9 g vs. saline-treated 38.8±1.0 g; P<0.01), 50.7% (nesfatin-1-treated 19.1±0.9 g vs. saline-treated 38.8±1.0 g, P<0.01) and 58.5% (nesfatin-1-treated 16.1±0.8 g vs. saline-treated 38.8±1.0 g, P<0.01) in the 15 pmol (n = 14), 25 pmol (n = 15) and 50 pmol (n = 10) nesfatin-1 treated groups, respectively, at the end of treatment. Fig. 3 showed the maps of injection sites in DVC for the feeding and body weight experiments.

### 2. The effect of nesfatin-1 on DVC glucosensing neurons *in vivo*

All discharges of neurons were recorded before, during, and after a glucose or nesfatin-1 injection. GE neurons and GI neurons were identified using micro-injection of glucose through a four-barrel glass microelectrode connected to a four-channel pressure injector.

**Figure 4:**
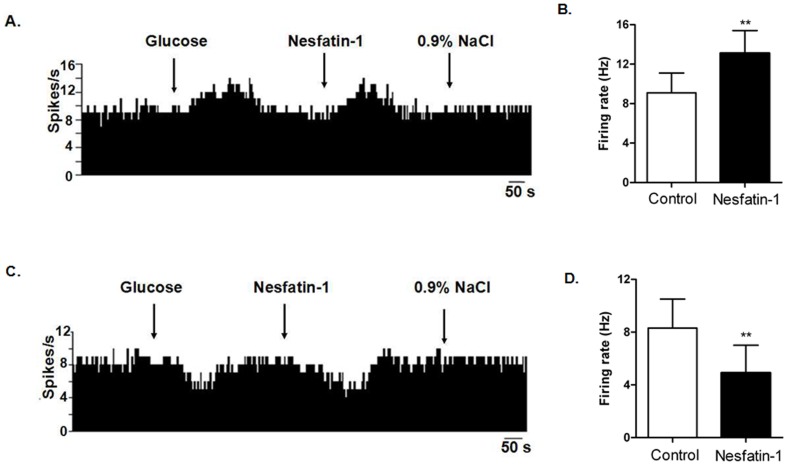
Effect of nesfatin-1 on glucosensing neurons in the dorsal vagal complex (DVC). (A). A GE-neuron was excited by nesfatin-1.The first arrow indicates 5 mM glucose, the second arrow indicates nesfatin-1 (1.5×10^−8^ M), and the third arrow indicates 0.9% NaCl-treated control. (B). Changes in firing rate after application of nesfatin-1 (n = 20, **P<0.01). (C). A GI-neuron was inhibited by nesfatin-1. (D). Changes in firing rate after application of nesfatin-1 (n = 33, **P<0.01).

After glucose was applied to the surface of 97 spontaneously active neurons recorded in 56 rats, 39 were identified as GI neurons and 26 as GE neurons. A control injection of 0.9% NaCl was used to rule out any neurons that responded to Na^+^ or Cl^−^ applications. Of 39 GI neurons in the DVC examined, 33 were depressed, 1 was activated, and 5 failed to respond to nesfatin-1 (Table 1). Administration of nesfatin-1 significantly decreased the firing rate of GI neurons from 8.3±2.2 Hz to 4.9±2.1 Hz (n = 33, P<0.01, Fig. 4). Of the 26 GE neurons, 20 were excited, 3 were inhibited, and 3 show no response to nesfatin-1. Micro-injection of nesfatin-1 significantly increased the spontaneous firing rate of GE neurons from 9.1±2.0 Hz to 13.1±2.3 Hz (n = 20, P<0.01).

**Table 1:** Effects of nesfatin-1 on glucosensing neurons in DVC.

Response to glucose	Response to nesfatin-1			
	Depressed	Activated	No response	Total
Depressed	33 (84.6%)	1 (2.6%)	5 (12.8%)	39 (100.0%)
Activated	3 (11.5%)	20 (77.0%)	3 (11.5%)	26 (100.0%)

### 3. The effect of nesfatin-1 on gastric distension sensitive neurons in DVC

**Figure 5:**
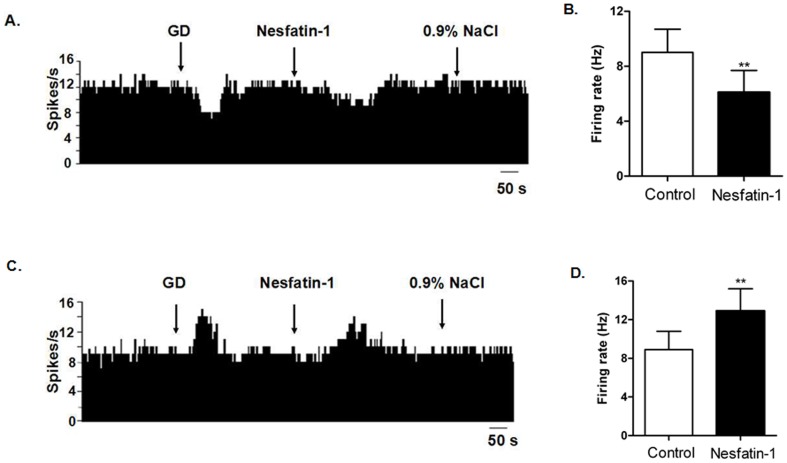
The effect of nesfatin-1 on gastric distension (GD) excitatory (GD-EXC) and inhibitory (GD-INH) neurons in the dorsal vagal complex (DVC). (A).A GD-INH neuron was inhibited by nesfatin-1. The first arrow indicates gastric distention, the second arrow indicates nesfatin-1 (1.5×10^−8^ M), and the third arrow indicates 0.9% NaCl-treated control. (B). Changes in firing rate after application of nesfatin-1 (n = 16, **P<0.01). (C). A GD-EXC neuron was excited by nesfatin-1. (D). Changes in firing rate after application of nesfatin-1 (n = 32, **P<0.01).

Of 60 GD-sensitive neurons in the DVC, 30.0% (18/60) were inhibited (GD-INH) and 70.0% (42/60) were excited (GD-EXC) (Table 2, Fig.5). Administration of nesfatin-1 decreased the firing rate of 16 GD-INH neurons from 9.0±1.7 Hz to 6.1±1.6 Hz (P<0.01). Of the 42 GD-EXC neurons tested with nesfatin-1, 32 were excited (from 8.9±1.9 Hz to 12.9±2.3 Hz, P<0.01), and 10 did not respond.

**Table 2:** Effects of nesfatin-1 on GD neurons in DVC.

Response to GD	Response to nesfatin-1			
	Depressed	Activated	No response	Total
Depressed	16 (88.9%)	0(0.0%)	2 (11.1%)	18(100.0%)
Activated	0 (0.0%)	32 (76.2%)	10 (23.8%)	42 (100.0%)

### 4. The effect of nesfatin-1 on DVC glucosensing neurons *in vitro*

**Figure 6:**
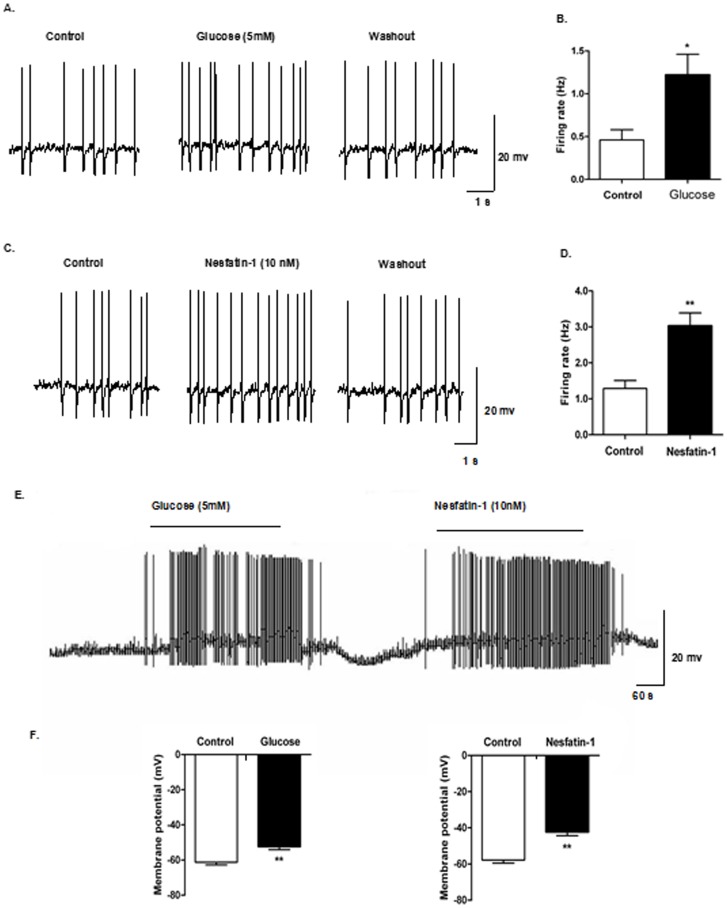
Nesfatin-1 increased excitability of GE-neurons in DVC. Current-clamp recordings from DVC neurons in slice preparation. (A) Identification of GE neurons in DVC. Representative raw traces of spontaneous action potentials were recorded before, during, and after application of glucose (5 mM), (B) Changes in firing rate after application of glucose. (C-D) Changes in firing rate after application of nesfatin-1 (10 nM). (E) Depolarizing responses to 5 mM glucose and 10 nM nesfatin-1 (horizontal bar). (F) Mean response to bath application of 5 mM glucose and 10 nM nesfatin-1.

A total of 43 spontaneously firing neurons were recorded in the DVC brain slices. To identify glucosensing neurons, 5 mM glucose was applied to the bath. Of these, 20 neurons were identified as GE neurons, as they increased their firing rate from 0.46±0.12 to 1.22±0.24 Hz and depolarized the membrane potential from -61.33±1.41 to −52.32±1.79 mV (Fig. 6). These GE neurons increased their action potential frequency from 1.29±0.22 to 3.03±0.36 Hz and their membrane potential from −57.18±1.71 to −42.25±2.06 mV in response to 10 nM nesfatin-1 (Table 3, Fig. 6).

**Table 3:** Effects of nesfatin-1 on glucosensing neurons in DVC *in vitro*.

Response to glucose	Response to nesfatin-1			
	Activated	Depressed	No response	Total
Activated	18 (90.0%)	0 (0.0%)	2 (10.0%)	20 (100.0%)
Depressed	0 (0.0%)	10 (90.9%)	1 (9.1%)	11 (100.0%)

**Figure 7:**
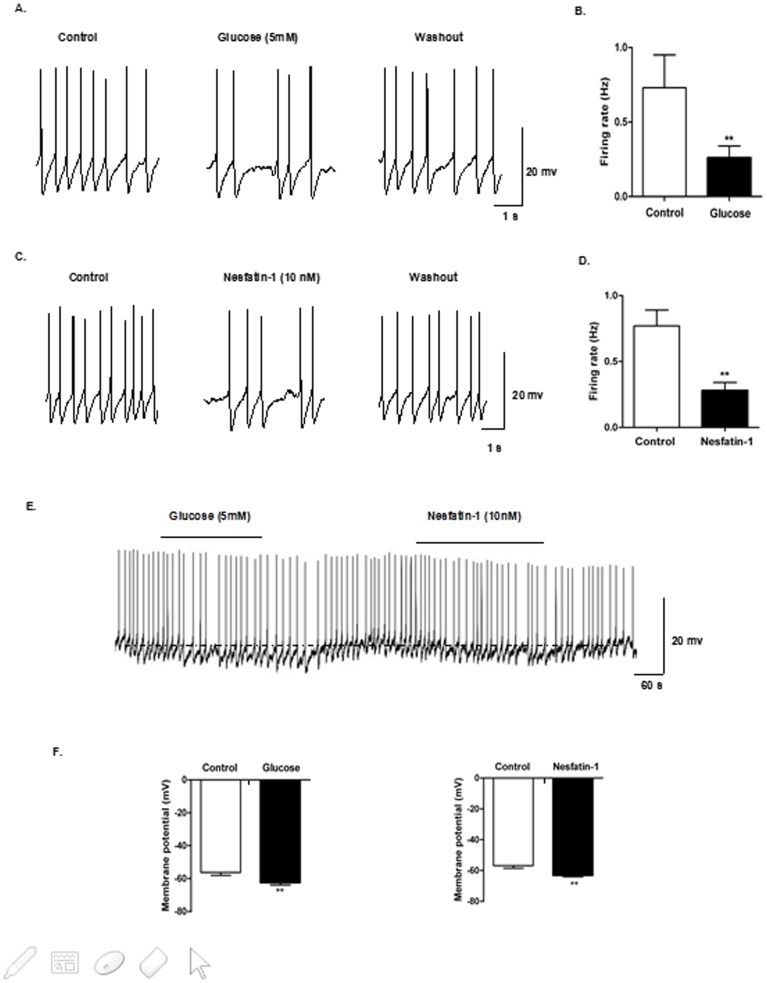
Nesfatin-1 decreased excitability of GI-neurons in DVC. Current-clamp recordings from DVC neurons in slice preparation. (A) Identification of GI neurons in the DVC. Representative raw traces of spontaneous action potentials were recorded before, during, and after application of glucose (5 mM), (B) Changes in firing rate after application of glucose. (C-D) Changes in firing rate after application of nesfatin-1 (10 nM). (E) Hyperpolarizing responses to 5 mM glucose and 10 nM nesfatin-1 (horizontal bar). (F) Mean response to bath application of 5 mM glucose and 10 nM nesfatin-1.

A total of 11 DVC neurons were identified as GI neurons, as they decreased firing frequency from 0.73±0.22 to 0.26±0.08 Hz and membrane potential from −56.27±1.78 to −62.44±1.48 mV. After perfusion with 10 nM nesfatin-1, GI neurons decreased their firing rate from 0.77±0.12 to 0.28±0.06 Hz and their membrane potential from −56.81±1.69 to −63.11±0.91 mV (Table 3, Fig. 7)

## Discussion

NUCB2/nesfatin-1 immunoreactivity has been detected recently in the DVC [2], including in the AP, NTS and DMNV. Furthermore, central or peripheral injections of nesfatin-1 increase c-Fos expression in NTS neurons [8], [19]. Although studies have focused on the role of nesfatin-1 in the hypothalamic nuclei regulating food intake, few have investigated the role of nesfatin-1 in the DVC. NUCB2 or nesfatin-1 (5 or 25 pmol) injected into the 3v inhibits nocturnal feeding in Wistar rats [1]. Stengel et al.[6] also shows that low doses of nesfatin-1 injected into the 4v at the hindbrain level (0.05 g/rat, 5 pmol) or into the cisterna magna (0.5 g/rat, 50 pmol) through a chronic cannula inhibits the first hour of dark-phase food intake and reduces cumulative food intake for 5 h after injection. In the present study, we extend these findings by showing that a low-dose injection of nesfatin-1 (15, 25, or 50 pmol/rat) into the DVC before the onset of the dark period significantly reduced the 5 h cumulative food intake in rats and that daily injections over a 10-day period reduced body weight gain. Thus, the DVC may be a critical site in addition to the PVN through which nesfatin-1 exerts its anorectic effects on food intake.

*In vivo* studies indicate that glucosensing neurons located in the hypothalamus and lower brainstem are involved in glucoprivic feeding and homeostatic control of blood glucose [12], [13], and they have recently received more attention for their potential role in appetite regulation [20]. To investigate the satiety-promoting mechanisms of nesfatin-1, we examined the effect of nesfatin-1 on the excitability of neurons in the DVC, one of the most prominent sites of nesfatin-1 expression, *in vivo* and *in vitro* [2], [3]. The results from *in vivo* experiment are generally consistent with the results of *in vitro*: nesfatin-1 (10 nM) increased the firing frequency of most GE neurons and decreased the firing rate of most GI neurons in both experiments. There appears to be a good consistency between the results obtained *in vivo* and *in vitro*. However, some little discrepancies do exist. The spontaneously firing rate of glucosensing neuron in brain slice was lower than that *in vivo*. Nesfatin-1 inhibited the firing rate of GI neurons by 40.9% *in vivo* and 63.6% *in vitro*, while nesfatin-1 excited the firing rate of GE neurons by 44.0% *in vivo* and 134.9% *in vitro*. Nesfatin-1 had more potent effects on glucosesing neurons in brain slice by perfusion than local action *in vivo*. The hypothesis that nesfatinergic neurons are stimulated by decreasing glucose levels is supported by a recent study by Bonnet et al. [11], who showed that insulin-activated neurons in the hypothalamus and DVC express nesfatin-1. Using Fluorogold as a retrograde tracer, Bonnet et al. [11] also shows that hypoglycemia-activated nesfatinergic neurons in the DMNV project to the stomach and pancreas, and that insulin selectively activates a fraction of nesfatin-1 neurons in the A2/C2 catecholaminergic hindbrain group [11]. This subpopulation of catecholamine neurons that is selectively activated by hypoglycemic stimuli plays a significant role in feeding [12], [13], [14]. As we all known, PVN has nesfatin-1 neurons projection to NTS. Although the firing rate recorded might be affected by nesfatin-1 released from PVN, yet the results from *in vitro* study eliminated the endogenous nesfatin-1 effects. Our data suggest that nesfatin-1 may partially control food intake by modulating the excitability of glucosensing neurons in the DVC.

Gastric mechanosensations regulate satiation during food intake [16]. GD activates mechanosensitive tension receptors, which relay the information through the vagal and splanchnic nerves to the DVC. GD significantly increases the number of c-Fos-positive cells that express NUCB2/nesfatin-1 in the NTS [21] and induces c-Fos expression in catecholaminergic and glucagon-like peptide-1/2-expressing neurons that modulate food intake in the vagal dorsal complex [18], [22]. Our data demonstrated that the GD-sensitive neurons responded to nesfatin-1. Bonnet et al. [21] also shows GD-induced activation of NTS NUCB2/nesfatinergic neurons in the brainstem neuronal pathways that modulate gastric functions in rats, indicating GD may inhibit food intake through NUCB2/nesfatin-1 neuron activation in the NTS.

In conclusion, nesfatin-1 may partially control food intake by modulating the excitability of glucosensing and GD-sensitive neurons in the DVC.
